# Two Cases of Secondary Anterior Perineal Hernia Repaired with a Pedicled Gracilis Muscle Flap

**DOI:** 10.70352/scrj.cr.26-0174

**Published:** 2026-06-17

**Authors:** Kentaro Chikaraishi, Katsuhito Suwa, Tomoyoshi Okamoto, Ken Eto

**Affiliations:** 1 Department of Surgery, The Jikei University West Medical Center, Komae, Tokyo, Japan; 2 Department of Surgery, The Jikei University Hospital, Tokyo, Japan

**Keywords:** anterior perineal hernia, gracilis muscle flap, pelvis, radical cystectomy

## Abstract

**INTRODUCTION:**

Secondary anterior perineal hernia (SAPH) is an extremely rare clinical entity. Due to the fragility of the posterior anchoring tissues, mesh repair carries a risk of late complications, such as rectal injury. Herein, we report 2 cases of male SAPH successfully repaired using a pedicled gracilis muscle flap.

**CASE PRESENTATION:**

**Case 1:** A man in his 70s developed a SAPH after undergoing a total cystectomy. Five years postoperatively, he presented with a bulging sensation in the perineal and scrotal regions, which caused significant discomfort, particularly when sitting. Physical examination revealed a protrusion extending from the base of the penis to the anus. Plain abdominal CT demonstrated herniation of the small intestine through a defect between the anus and the pubic bone, leading to a diagnosis of SAPH. The hernial sac was circumferentially dissected via a transperineal approach with the patient in the lithotomy position. A pedicled gracilis muscle flap was harvested from the left thigh, transposed through a subcutaneous tunnel, and packed into the hernial orifice. The flap was secured to the pubis, right ischium, and superficial transverse perineal muscle. No recurrence has been observed 3 months postoperatively. **Case 2:** A man in his 80s developed a hernia 1 year after a total cystectomy. Similar to Case 1, the hernia was repaired using a left gracilis muscle flap. No recurrence has been noted at the 15-month follow-up.

**CONCLUSIONS:**

Repair using a pedicled gracilis muscle flap is an effective procedure that avoids mesh-related complications.

## Abbreviations

APR
: abdominoperineal resection
SAPH
: secondary anterior perineal hernia
SSI
: surgical site infection

## INTRODUCTION

Perineal hernia is a rare clinical entity characterized by the protrusion of intra-abdominal contents through the pelvic floor. It is classified into primary (congenital or acquired) and secondary types. Primary congenital perineal hernia is thought to result from the failure of the embryonic cul-de-sac to regress,^1)^ while primary acquired perineal hernia is associated with factors such as pregnancy, obesity, chronic ascites, and chronic pelvic floor infections.^1)^ Secondary perineal hernia, a subtype of incisional hernia, typically follows major pelvic surgeries such as APR or radical cystectomy.^1)^ Reported risk factors for secondary perineal hernia include female sex, previous hysterectomy, previous coccygectomy, a history of radiation therapy, redundant small bowel mesentery, perineal wound infection, non-closure of the pelvic peritoneum, extralevator APR, and smoking history.^2–4)^ While conservative management is appropriate for asymptomatic cases, surgical intervention is indicated for patients experiencing discomfort, pain, or urinary dysfunction associated with perineal bulging.^5)^

The majority of reported cases of secondary perineal hernia are posterior hernias following procedures such as APR or pelvic exenteration. These typically develop within 1 year postoperatively,^2)^ with a reported incidence ranging from 1% to 26%.^2,6)^ In contrast, SAPH is extremely rare; to date, only a single case of SAPH in a female patient treated with mesh repair has been reported.^3)^

Anatomically, the repair of SAPH presents a significant challenge because there are no robust structures other than the superficial transverse perineal muscle available for posterior fixation. Furthermore, the tissue plane between this muscle and the rectum is thin, posing a risk of inadvertent rectal injury or mesh-related complications if prosthetic materials are fixed directly to this area. Therefore, we performed a novel repair using a pedicled gracilis muscle flap for SAPH in 2 male patients. To the best of our knowledge, this is the first report of this surgical approach for SAPH in males that achieved favorable clinical outcomes.

## CASE PRESENTATION

**Fig. 1:**
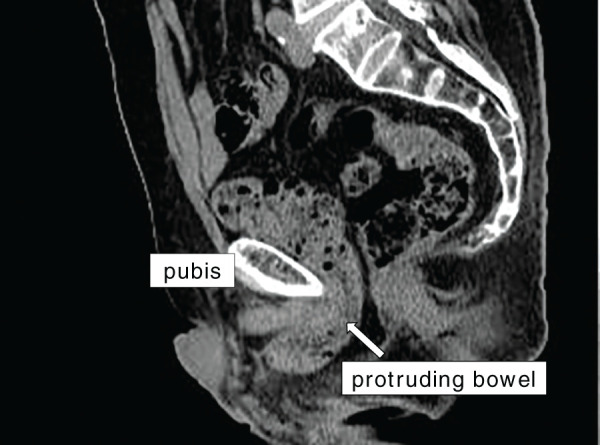
Preoperative CT scan of Case 1. Small bowel herniation is observed anterior to the superficial transverse perineal muscle, leading to the diagnosis of SAPH. SAPH, secondary anterior perineal hernia

Case 1 was a man in his 70s who had undergone radical cystectomy with ileal conduit urinary diversion for bladder cancer 5 years prior. He had remained recurrence-free since the initial surgery. Five years postoperatively, he presented with a bulging sensation in the perineal and scrotal regions, which caused significant discomfort, particularly when sitting. Physical examination revealed a protrusion extending from the base of the penis to the anus. Plain abdominal CT demonstrated herniation of the small intestine through a defect between the anus and the pubic bone, leading to a diagnosis of SAPH (**Fig. 1**).

**Fig. 2:**
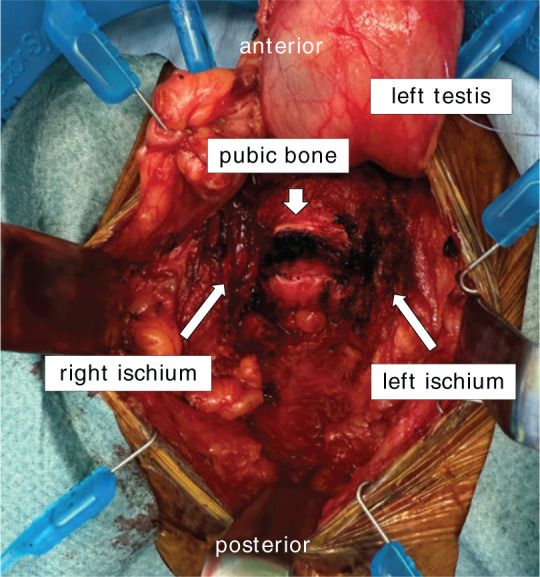
Intraoperative findings in the lithotomy position. The surgical field was dissected to adequately expose the posterior aspect of the pubic bone (anteriorly), the superficial transverse perineal muscle (posteriorly), and the ischium and pubococcygeus muscles (laterally).

The procedure was performed with the patient in the lithotomy position. A skin incision was made directly over the hernia sac to facilitate its identification. Given the proximity of the testes to the sac, circumferential adhesiolysis was performed with meticulous care to avoid injury. Dissection was continued until the posterior aspect of the pubic bone anteriorly, the superficial transverse perineal muscle posteriorly, and the ischium and pubococcygeus muscles laterally were clearly visualized (**Fig. 2**). The hernia orifice measured 25 × 25 mm.

**Fig. 3:**
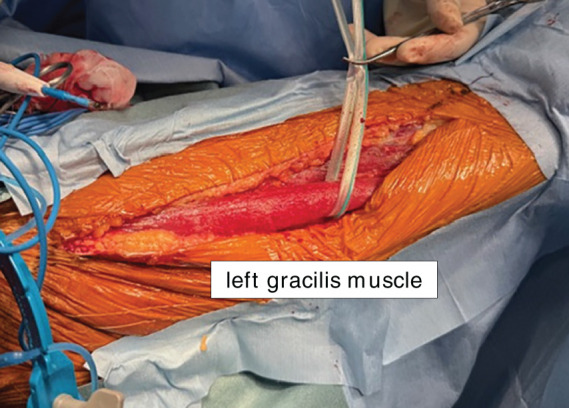
Harvest of the gracilis muscle flap. The left gracilis muscle was identified and transected to create a pedicled muscle flap.

**Fig. 4:**
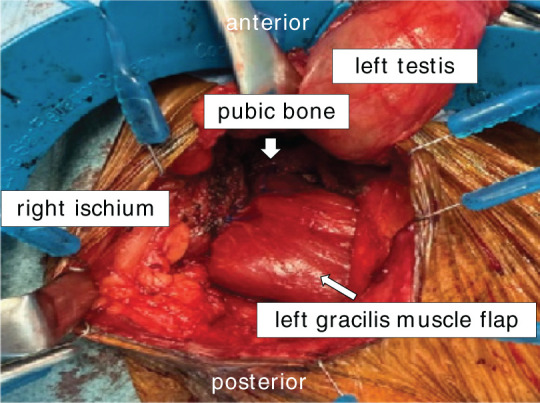
Transposition of the muscle flap. The gracilis muscle flap was transposed through a subcutaneous tunnel and used to fill the hernia orifice.

Subsequently, while maintaining the lithotomy position, we proceeded with harvesting the gracilis muscle flap. Plastic surgeons performed a skin incision on the medial aspect of the left thigh, and the gracilis muscle was dissected as far distally as possible before being transected (**Fig. 3**). The muscle flap, with its vascular pedicle preserved, was rotated and transposed to the hernia orifice through a subcutaneous tunnel. The flap was first anchored to the left ischium with 3 interrupted 2-0 Prolene sutures (Ethicon, Somerville, NJ, USA). After filling the hernia orifice with the pedicled gracilis flap, it was sequentially anchored to the pubic bone and the right ischium, and sutured to the superficial transverse perineal muscle with interrupted 2-0 Prolene sutures (**Fig. 4**). During posterior fixation, care was taken to ensure that the suture depth was not excessive to avoid injury to the rectum. Closed suction drains were placed in both the perineal and left thigh wounds before closure.

**Fig. 5:**
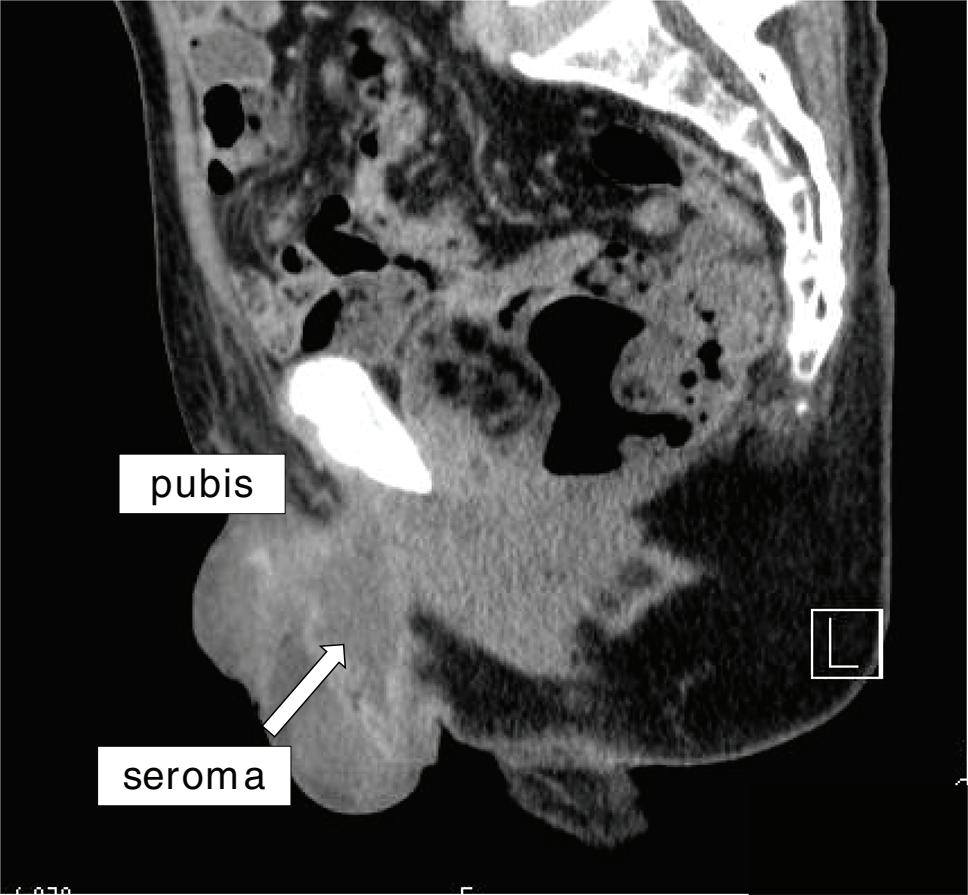
Postoperative contrast-enhanced CT of Case 1 at 3 months. The muscle flap demonstrates excellent blood supply (enhancement), and there is no evidence of hernia recurrence.

The total operative time was 218 min, with minimal blood loss. The postoperative course was uneventful, and the patient was discharged on POD 12. At 3 months postoperatively, there was no evidence of recurrence, although an asymptomatic seroma was identified and managed conservatively (**Fig. 5**).

Case 2 was a man in his 80s who had undergone robot-assisted radical cystectomy with ileal conduit urinary diversion and total urethrectomy 2 years prior. Approximately 1 year postoperatively, he noticed a bulging sensation in the perineal region, and this discomfort had gradually progressed. Physical examination revealed a protrusion extending from the pubic bone to the anus. CT demonstrated the herniation of intestinal loops into the scrotum through a pelvic floor defect, leading to a diagnosis of SAPH.

The procedure was performed in the lithotomy position. A skin incision was made directly over the hernia sac and the sac was identified. After circumferential adhesiolysis, dissection was continued until the posterior aspect of the pubic bone anteriorly, the superficial transverse perineal muscle posteriorly, and the ischium and pubococcygeus muscles laterally were sufficiently exposed. The hernia orifice measured 20 × 20 mm.

With the patient remaining in the lithotomy position, we proceeded to harvest the gracilis muscle flap. Plastic surgeons performed a skin incision on the medial aspect of the left thigh, and the gracilis muscle was dissected as far distally as possible before being transected. The muscle flap was then rotated with its vascular pedicle intact and transposed to the hernia orifice through a subcutaneous tunnel. The flap was first anchored to the left ischium with 3 interrupted 2-0 Prolene sutures. After filling the hernia orifice with the flap, it was sequentially fixed to the pubic bone, the right ischium, and sutured to the superficial transverse perineal muscle with interrupted 2-0 Prolene sutures. Closed suction drains were placed in both the perineal and left thigh wounds.

**Fig. 6:**
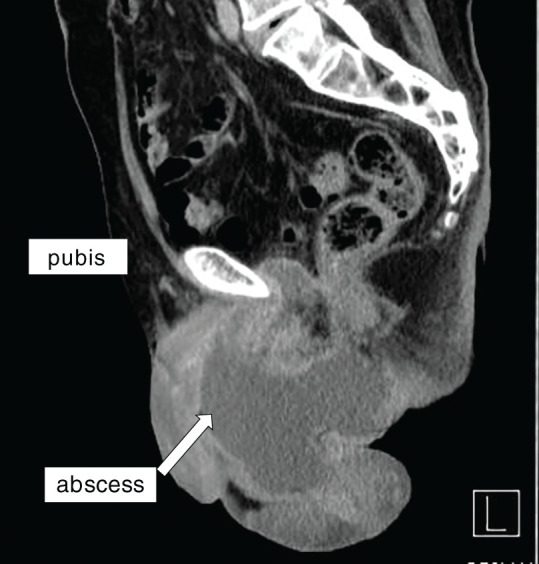
In Case 2, the patient presented with perineal swelling. CT revealed abscess formation, which was treated with incision and drainage.

The total operative time was 265 min, with minimal blood loss. The patient was discharged on POD 10. However, on POD 12, he returned to our hospital complaining of perineal swelling (**Fig. 6**). Based on a diagnosis of SSI, incision and drainage were performed. The symptoms resolved immediately following the drainage procedure. No recurrence has been observed as of 15 months postoperatively.

## DISCUSSION

Surgical approaches for perineal hernia repair are generally classified as transabdominal and transperineal, with reconstructive techniques categorized into mesh repair, flap reconstruction, and primary tissue suture repair. The transabdominal approach provides a comprehensive view of the hernia orifice, facilitating a wider overlap of the repair material; however, it necessitates intra-abdominal adhesiolysis, which carries a potential risk of visceral injury.^7)^ Conversely, while the transperineal approach allows for direct and easy access to the hernia orifice, the surgical field is often more confined, and it is associated with a higher incidence of wound-related complications, reported to be as high as 28%.^8)^ We selected the transperineal approach because it allows us to perform both the gracilis muscle flap harvest and its transposition into the hernia orifice via a subcutaneous tunnel, thereby avoiding additional extensive incisions.

Outcomes of repair for posterior perineal hernia vary significantly across the literature. In posterior repairs, prosthetic mesh or muscle flaps are anchored laterally to the residual levator ani muscle or the ischial tuberosity, and posteriorly to the coccyx or the coccygeus muscle. However, the lack of “good stuff”—robust anatomical structures for anterior fixation—often contributes to high recurrence rates.^9)^ Junsheng and colleagues reported recurrence rates of 21.9% and 14.3% for mesh and flap repairs, respectively, in secondary perineal hernia.^4)^ In contrast, Sarah and colleagues observed recurrences in 37% of mesh repairs compared to only 9% in flap repairs.^10)^ While primary tissue suture repair is associated with a markedly higher recurrence rate of approximately 50%,^5)^ a gold standard technique has yet to be established, and clinical consensus remains elusive. Furthermore, many existing case reports are characterized by short follow-up periods, leaving the definitive long-term surgical outcomes unclear.^5)^

The challenge of identifying adequate fixation structures also applies to SAPH. While anterior fixation to the pubic bone and lateral fixation to the residual levator ani muscle and ischium are feasible, the posterior aspect lacks any robust tissue for anchoring other than the superficial transverse perineal muscle. However, due to the thin boundary between the superficial transverse perineal muscle and the rectum, direct fixation of mesh to the rectum or long-term contact between the prosthetic material and the rectal wall poses a significant concern. Therefore, the use of synthetic mesh or other prosthetics requires extreme caution in this anatomical region.

Although clinical data supporting the avoidance of direct contact between plastic mesh and the intestinal wall have historically been limited, complications associated with laparoscopic ventral rectopexy—a procedure involving direct suturing of mesh to the rectum for rectal prolapse—provide critical insight. Reported late complications of this procedure include mesh erosion (2.0%–3.7%) and rectovaginal fistula (0.3%–0.5%), sometimes necessitating partial mesh excision (1.0%–1.6%) or even stoma creation (0.1%).^11,12)^ Furthermore, in the Sugarbaker technique for parastomal hernia repair, complications such as bowel injury, necrosis, fistula formation, intestinal obstruction, and infection resulting from contact between the mesh and the bowel have been reported.^13)^ Consequently, a similar risk of late complications may exist when using mesh for SAPH repair, necessitating prudent surgical judgment. Although Alvarez Garzón et al. reported a case of SAPH successfully treated with mesh repair,^3)^ as previously noted, there is currently a lack of data regarding the long-term complications of this approach in the context of SAPH.

In the present cases, we performed hernia repair using a pedicled gracilis muscle flap to avoid the use of synthetic mesh. Given the technical complexity of dissecting the gracilis muscle while preserving its blood supply, plastic surgeons who are adept at managing both the surgical defect and the patient’s underlying comorbidities play an essential role in optimizing outcomes.^14)^ In patients with conditions often involving a stoma or ileal conduit, the gracilis muscle flap is an effective option for reconstructing perineal defects, offering an alternative to the rectus abdominis myocutaneous flap.^8)^ While harvesting the gracilis muscle results in an 11% reduction in adductor muscle strength, this loss is generally not clinically significant.^8)^ Furthermore, although sensory loss in the territory of the cutaneous branch of the obturator nerve occurs in 40.5% of cases, medical intervention is rarely required.^8)^ In perineal hernia repairs using the gracilis muscle, the reported incidence of SSI is 12%, with some cases requiring debridement or percutaneous drainage.^8)^ Other potential complications associated with pedicled gracilis flaps include impaired blood flow or necrosis due to vascular pedicle torsion, and flap atrophy following denervation, which may contribute to recurrence.^15)^ In our patients, no postoperative motor or sensory deficits were observed. Contrast-enhanced CT performed 1 year postoperatively in Case 2 demonstrated excellent enhancement of the muscle flaps, with no evidence of flap atrophy or hernia recurrence. The gracilis muscle flap provided adequate volume to fill the defect relative to the size of the SAPH hernial orifice. Furthermore, although Case 2 developed an SSI necessitating incision and drainage, the avoidance of mesh and the maintenance of a robust blood supply within the muscle flap likely contributed to the effective control of the infection.^8)^ The rich vascularization of the muscle flap likely facilitated the management of the SSI, preventing the persistent infections often associated with prosthetic materials. This clinical observation underscores the safety advantage of using autologous tissue, particularly in the perineal region where the risk of wound-related complications is inherently high.

A potential limitation of this technique is that muscle flaps may possess less long-term structural integrity compared to synthetic mesh.^10)^ Because the pelvic floor is subjected to persistent intra-abdominal pressure over time, whether a muscle flap provides sufficient durability remains to be fully elucidated, and long-term outcomes have not yet been adequately investigated. Large-scale prospective studies are warranted to establish the optimal surgical approach for this condition.

## CONCLUSIONS

In the present cases, we performed repair using an autologous gracilis muscle flap specifically to avoid the risks associated with fixing synthetic mesh directly to the rectal wall. Our experience suggests that this technique is a valuable surgical option for SAPH, particularly in clinical scenarios where the use of prosthetic materials is considered unfavorable.

## DECLARATIONS

### Availability of data and materials

The datasets supporting the conclusions of this article are included within the article.
